# Diffusion Tensor Imaging Revealed Microstructural Changes in Normal-Appearing White Matter Regions in Relapsing–Remitting Multiple Sclerosis

**DOI:** 10.3389/fnins.2022.837452

**Published:** 2022-03-02

**Authors:** Jianfeng Bao, Hui Tu, Yijia Li, Jubao Sun, Zhigang Hu, Fengshou Zhang, Jinghua Li

**Affiliations:** ^1^College of Medical Technology and Engineering, Henan University of Science and Technology, Luoyang, China; ^2^Department of Magnetic Resonance Imaging, The First Affiliated Hospital of Zhengzhou University, Zhengzhou University, Zhengzhou, China; ^3^MRI Center, The First Affiliated Hospital of Henan University of Science and Technology, Luoyang, China

**Keywords:** relapsing-remitting multiple sclerosis, normal-appearing white matter, diffusion tensor imaging, magnetic resonance imaging, white matter tracts atlas, probabilistic lesion map

## Abstract

**Background:**

Axons and myelin sheaths are the physical foundation for white matter (WM) to perform normal functions. Our previous study found the metabolite abnormalities in frontal, parietal, and occipital normal-appearing white matter (NAWM) regions in relapsing–remitting multiple sclerosis (RRMS) patients by applying a 2D ^1^H magnetic resonance spectroscopic imaging method. Since the metabolite changes may associate with the microstructure changes, we used the diffusion tensor imaging (DTI) method to assess the integrity of NAWM in this study.

**Method:**

Diffusion tensor imaging scan was performed on 17 clinically definite RRMS patients and 21 age-matched healthy controls on a 3.0-T scanner. DTI metrics including fractional anisotropy (FA), mean diffusivity (MD), axial diffusivity (AD), and radial diffusivity (RD) were extracted from 19 predefined regions of interest (ROIs), which were generated by removing a mask of manually drawn probabilistic lesion map from the Johns Hopkins University white-matter atlas. The mean values of FA, MD, AD, and RD were compared between different groups in the same ROIs.

**Results:**

A probabilistic lesion map was successfully generated, and the lesion regions were eliminated from the WM atlas. We found that the RRMS patients had significantly lower FA in the entire corpus callosum (CC), bilateral of anterior corona radiata, and right posterior thalamic radiation (PTR). At the same time, RRMS patients showed significantly higher MD in the bilateral anterior corona radiata and superior corona radiata. Moreover, all AD values increased, and the bilateral external capsule, PTR, and left tapetum NAWM show statistical significance. What is more, all NAWM tracts showed increasing RD values in RRMS patients, and the bilateral superior corona radiata, the anterior corona radiata, right PTR, and the genu CC reach statistical significance.

**Conclusion:**

Our study revealed widespread microstructure changes in NAWM in RRMS patients through a ready-made WM atlas and probabilistic lesion map. These findings support the hypothesis of demyelination, accumulation of inflammatory cells, and axonal injury in NAWM for RRMS. The DTI-based metrics could be considered as potential non-invasive biomarkers of disease severity.

## Introduction

Multiple sclerosis (MS) is an autoimmune disease affecting the central nervous system (CNS). Two hundred million people have been suffering from multiple physical and psychological symptoms caused by MS (Hauser and Cree, 2020). The clinical symptoms of MS include limb weakness, sensory disorder, blurred vision, cognitive impairment, and dizziness, which may significantly impair the quality of life in people with MS (Filippi et al., 2020). Although the pathogenesis of MS is still not fully understood, much evidence has been pointing to the chronic inflammatory demyelination of white matter (WM). Thus, MRI has been extensively used to scan the MS patient for clinical purposes, including diagnosis, monitoring progression, and evaluating treatment outcomes. Besides clinical routine T1W, T2W, and diffusion-weighted images, more advanced MR imaging techniques have been applied to explore the functions and microstructures of the CNS of MS (Dal-Bianco et al., 2021). Advanced MRI techniques will detect different physiological signals and provide various pathological information using various post-processing approaches, thus they may improve our understanding of the pathological specificity and better reveal the relationship between anatomical damage and phenotypes.

Diffusion tensor imaging (DTI), with a bipolar pair of diffusion gradients, is very sensitive to the movement of water molecules. Only signals from the movement of water molecules along the diffusion gradients can be refocused and detected; other signals will lose phase by the diffusion gradients. DTI is a powerful technique for characterizing WM microstructural alteration by calculating the metrics of fractional anisotropy (FA), mean diffusivity (MD), axial diffusivity (AD), and radial diffusivity (RD). DTI has been widely applied to characterize WM alterations in MS (Andersen et al., 2018). Combined with T1 or gadolinium-enhancing images, lower FA and higher MD values may suggest the severity of WM damage in the focal lesions, and the DTI metrics even have the potential to grade and predict the development of the acute lesions of MS (Werring et al., 1999; Bammer et al., 2000; Filippi et al., 2001). Moreover, a recent 4-year study found that the diffusivity metrics of corpus callosum (CC) can provide additional information to monitor individual disability progression (Kolasa et al., 2019). Taking the advantages of various DTI analysis methods, increased MD and decreased FA values were extensively observed in NAWM of MS patients, and the severity increased from far away to near the locations of the existing lesions (Cercignani et al., 2001; Hasan et al., 2005). The abnormalities reflect the early stages of MS and may contain the formulation of new lesions. Besides the brain structure, brain metabolite changes were also observed by ^1^H magnetic resonance spectroscopy (MRS) in MS patients. Recently, including our study (Sun et al., 2017), several studies have reported the decreases in NAA and NAA/Cr ratio in lesions and in normal-appearing tissues in MS patients (De Stefano et al., 2005), and significant correlations between the metabolic ratio of NAA/Cr and clinical disability were observed (MacMillan et al., 2015; Cao et al., 2018).

Since only a modest correlation has been observed between the WM lesions and the clinical disabilities for MS patients, more and more researchers have started to study the normal-appearing white matter (NAWM) to explore the potential pathological mechanisms of MS. Previous studies reported numerous alterations in magnetization transfer ratio (MTR) (Vrenken et al., 2007), diffusion metrics (Werring et al., 2000), relaxation times (Stevenson et al., 2000), perfusion properties (Sowa et al., 2015), and metabolic characteristics (Rooney et al., 1997) in NAWM. Histopathological changes in NAWM depend on the distances to the lesions, and the near WM axon is more susceptible than that of distant WM found by MTR and post-mortem immunohistochemically analysis (Moll et al., 2011).

In summary, it is evident that various advanced MR imaging modalities or techniques can characterize different pathology profiles of the MS, not only providing isolated perspectives but indeed complementary information. Following our previous work using MRS (Sun et al., 2017), we now reported the DTI results. The primary aim of this work was to measure the NAWM microstructure changes in frontal, parietal, and occipital NAWM tracts between clinically definite relapsing–remitting multiple sclerosis (RRMS) patients and age-matched healthy controls. The secondary aim is to provide insights into the relationships between brain metabolism and NAWM microstructure.

## Materials and Methods

### Participants

In total, 38 individuals, 17 patients (11 female and 6 male, average age = 42.3 ± 13.2) with RRMS and 21 healthy controls (17 female and 4 male, age = 46.7 ± 8.9), were enrolled in the current study at the First Affiliated Hospital of Henan University of Science and Technology. All subjects have signed consent forms before participating in this study. This study was approved by the Local Institutional Ethics Committee on human research of the First Affiliated Hospital and College of Clinical Medicine of Henan University of Science.

### MRI Data Acquisition

The MRI data were obtained from a 3.0-T Philip MR scanner with a 16-channel head coil. T2-weighted fluid-attenuated inversion recovery (FLAIR) image was obtained for lesion regions of interest (ROIs) drawing with the following parameters: repetition time (TR) = 6,000 ms, echo time (TE) = 140 ms, inversion time (IR) = 2,000 ms, slice thickness = 7.5 mm, field of view (FOV) = 250 mm × 250 mm, and resolution matrix = 256 × 256. For DTI protocol, single-shot echo planar pulse sequence accelerated by sensitivity encoding (SENSE) parallel technique was applied with TR = 8,000 ms, TE = 101 ms, *b*-value = 0 and 1,000 s/mm^2^, and 30 gradient directions. Standard corona 3D inversion recovery T1 turbo field echo (3D IR-T1TFE) structure image was also acquired with the following parameters: isotropic voxel size of 0.94 mm × 0.94 mm × 0.94 mm, TI = 1,123 ms, flip angle = 6, and SENSE accelerated mode as well. All data were visual quality checked after the scan.

### Diffusion Tensor Imaging Pre-processing

The raw DICOM-format data were converted into NIfTI format using MRIcron.^1^ Then the brain skull was removed using a brain extraction tool in FMRIB Software Library FSL (v5.0.9^2^). Afterward, eddy current correction, motion correction, and distortion correction were performed in order for all individual DTI volumes. Then the diffusion tensor metrics were fitted at each voxel in native space using DTIFit in FSL. T1W images were registered to MIN_152 standard 2-mm brain images using non-linear registration. The T2 FLAIR image was registered to T1W images using linear image registration, and the transformed matrix was saved. The WM regions provided in JHU white-matter atlas in the standard space were inversely registered to native T1W structural space and then to the diffusion space for analysis.

### Data Processing

According to previous similar research, the lesion regions in MS patients were removed from this analysis through a probabilistic lesion map (Cai et al., 2016; Guo et al., 2019). The binary mask of probabilistic lesion map was generated to eliminate effects from the obvious lesions in this study. First, the lesion regions were manually labeled in the T1W image for each patient by two radiologists with 10 years’ experience, and all lesion regions were then registered to standard space through non-linear registration. After that, all regions are added together and then divided by the number of patients to obtain the probabilistic lesion map. Finally, the map was inversely registered to the native space, and a threshold of 0.3 was used to binarize this exclusion mask. The integrity and damage severity of the NAWM depends on multiple factors, and the focal lesions distribute widely in the brain. Thus, to avoid subjective judgment by artificial selection, the initial WM ROIs are from a public atlas (JHU white-matter atlas), and finally the subtractive WM tracts with number of voxels less than 20 were removed.

### Statistical Analysis

All statistical analysis was performed by SPSS (version 18.0; SPSS Inc., Chicago, III, United States). An independent-samples *t*-test was used to compare the DTI metrics in NAWM between RRMS patients and healthy controls. *P*-value < 0.01 was considered as significance level, and *p*-value < 0.001 was considered as highly significant. The Benjamini–Hochberg method was used to control the false discovery rate (FDR).

## Results

There were no statistical differences between patients and controls for ages and gender. All patients were diagnosed with RRMS according to McDonald’s criteria (Chiaravalloti et al., 2005) and routine MRI examinations.

**FIGURE 1:**
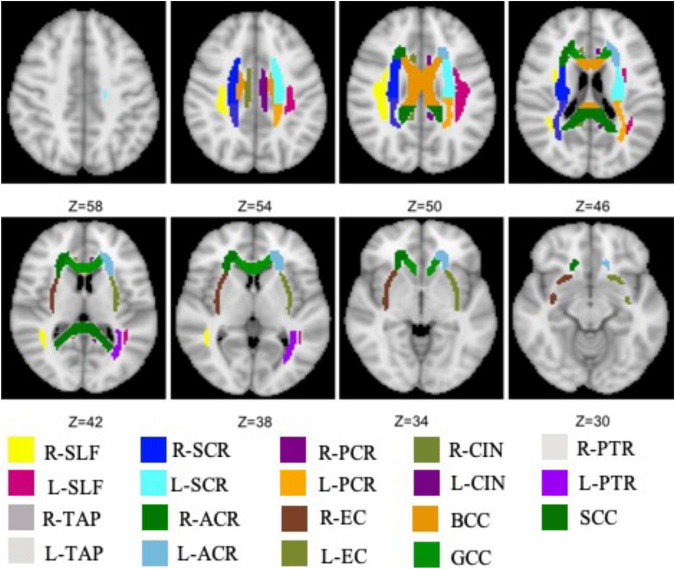
All WM tracts, extracted from JHU white-matter atlas, are analyzed in this work. SCR, superior corona radiata; SLF, superior longitudinal fasciculus; ACR, anterior corona radiata; PCR, posterior corona radiata; BCC, body of the corpus callosum; CIN, cingulate; EC, external capsule; GCC, genu of the corpus callosum; PTR, posterior thalamic radiation; SCC, splenium of the corpus callosum; TAP, tapetum.

**FIGURE 2:**
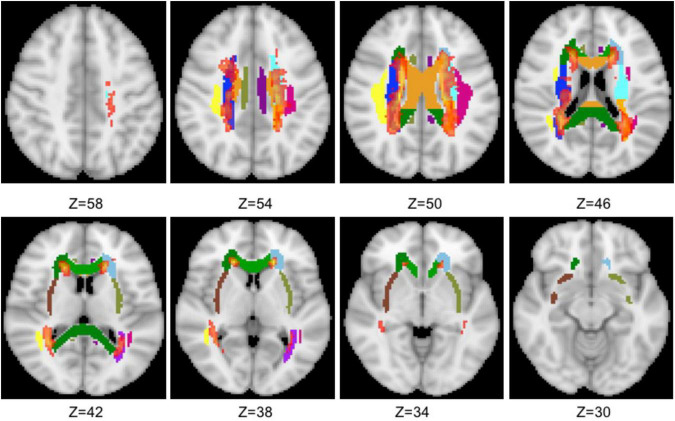
Overlaying the probabilistic lesion map (hot color, threshold 0.3) on the NAWM tracts. The other colors represent the same WM tracts as in Figure 1.

The total 19 WM tracts are shown in Figure 1 with different colors, including frontal (anterior corona radiata and genu of CC), parietal (superior longitudinal fasciculus, body of CC, cingulum, superior corona radiata, and external capsule), and occipital [posterior corona radiata, posterior thalamic radiation (PTR), splenium of CC, and tapetum] three NAWM regions. The probabilistic lesion map is shown in Figure 2 with hot color overlaying on the predefined WM tracts. It should be noticed that the posterior corona radiata and right tapetum were not used for DTI metrics comparisons because the number of the residual volume is less than 20.

As shown in Table 1, all the ROIs show reduced mean FA values in RRMS patients. And significantly lower FA values were observed in RRMS patients in six NAWM tracts, including the entire CC bilateral ACR and right PTR. For the MD, all NAWM tracts show higher value for RRMS patients than for controls, and five NAWM tracts, including ACR and SCR in both hemispheres were observed significant elevated.

**TABLE 1:** Comparison of DTI metrics of FA and MD in NAWM tracts between RRMS patients and controls using two-tailed Student’s *t*-test.

Location	Side	FA (mean ± SD)		MD (mean ± SD) 10^–3^ mm^2^ s^–1^	
		RRMS	Control	RRMS	Control
SCR	Left	0.3816 ± 0.0375	0.4307 ± 0.0577	0.7769 ± 0.0664**	0.6785 ± 0.0199
SCR	Right	0.3918 ± 0.0331	0.4824 ± 0.0450	0.7976 ± 0.0731**	0.6954 ± 0.0429
SLF	Left	0.3418 ± 0.0221	0.3769 ± 0.0386	0.8127 ± 0.0618	0.7523 ± 0.0380
SLF	Right	0.3377 ± 0.0352	0.3871 ± 0.0458	0.8106 ± 0.0510	0.7611 ± 0.0333
ACR	Left	0.3425 ± 0.0400**	0.3975 ± 0.0200	0.8397 ± 0.0716**	0.7418 ± 0.0226
ACR	Right	0.3390 ± 0.0393**	0.4034 ± 0.0292	0.7403 ± 0.0817**	0.8474 ± 0.0279
BCC	–	0.3831 ± 0.0389**	0.4503 ± 0.0334	1.0156 ± 0.1421	0.9606 ± 0.1271
CIN	Left	0.2707 ± 0.0423	0.3045 ± 0.0514	0.8690 ± 0.0659	0.8140 ± 0.0377
CIN	Right	0.2642 ± 0.0381	0.0304 ± 0.0460	0.8361 ± 0.0586	0.7950 ± 0.0510
EC	Left	0.3295 ± 0.0221	0.3423 ± 0.0205	0.8242 ± 0.0569	0.7698 ± 0.0392
EC	Right	0.3294 ± 0.0213	0.3331 ± 0.0191	0.8322 ± 0.0504	0.7775 ± 0.0243
GCC	–	0.3549 ± 0.0423**	0.4227 ± 0.0369	1.1287 ± 0.0906	1.0199 ± 0.0898
PTR	Left	0.4035 ± 0.0614	0.4472 ± 0.0318	0.945 ± 0.1245	0.8181 ± 0.0534
PTR	Right	0.3930 ± 0.0508*	0.4534 ± 0.0266	0.8538 ± 0.0974	0.7432 ± 0.0832
SCC	–	0.4159 ± 0.0464**	0.5087 ± 0.0393	1.1251 ± 0.1456	1.0363 ± 0.0937
TAP	Left	0.3685 ± 0.0540	0.3703 ± 0.0965	1.2971 ± 0.2899	1.5991 ± 0.3597

Significant differences of FA and MD between RRMS patients and healthy controls are shown by FDR adjusted *p < 0.01 and highly significant differences by **p < 0.001.

As shown Table 2, RRMS patients show elevated AD and RD values for all NAWM tracts. For AD, there are five regions show significantly higher values for RRMS patients than for controls, and they are PTR, EC in both hemispheres and left TAP. For RD, there are six regions that show significantly higher values for RRMS patients than for controls, and they are ACR, SCR and in both hemispheres, GCC, and right PTR.

**TABLE 2:** Comparison of DTI metrics of AD and RD in NAWM tracts between MS patients and controls using two-tailed Student’s *t*-test.

Location	Side	AD (mean ± SD) 10^–3^ mm^2^ s^–1^		RD (mean ± SD) 10^–3^ mm^2^ s^–1^	
		RRMS	Control	RRMS	Control
SCR	Left	1.1090 ± 0.0726	1.0465 ± 0.0528	0.6109 ± 0.0662**	0.4945 ± 0.0352
SCR	Right	1.1312 ± 0.0782	1.0353 ± 0.0903	0.6307 ± 0.0750**	0.5253 ± 0.0467
SLF	Left	1.0892 ± 0.0517	1.0523 ± 0.0382	0.6742 ± 0.0696	0.6018 ± 0.0496
SLF	Right	1.0980 ± 0.0577	1.0650 ± 0.0247	0.6667 ± 0.0504	0.6090 ± 0.0451
ACR	Left	1.1471 ± 0.0689	1.0733 ± 0.0285	0.6857 ± 0.0772**	0.5761 ± 0.0246
ACR	Right	1.1546 ± 0.0902	1.0812 ± 0.0258	0.6938 ± 0.0828**	0.5700 ± 0.0341
BCC	–	1.4285 ± 0.1643	1.4462 ± 0.1588	0.8091 ± 0.1362	0.7179 ± 0.1157
CIN	Left	1.1032 ± 0.0565	1.0712 ± 0.0486	0.7517 ± 0.0776	0.6850 ± 0.0520
CIN	Right	1.0569 ± 0.0522	1.0430 ± 0.0430	0.7258 ± 0.0680	0.6708 ± 0.0640
EC	Left	1.1107 ± 0.0595*	1.0496 ± 0.0404	0.6807 ± 0.0576	0.6296 ± 0.0410
EC	Right	1.1239 ± 0.0542**	1.0536 ± 0.0306	0.6865 ± 0.0510	0.6394 ± 0.0244
GCC	–	1.5264 ± 0.0845	1.4680 ± 0.0957	0.9300 ± 0.0995*	0.7960 ± 0.0937
PTR	Left	1.3435 ± 0.1189**	1.2040 ± 0.0593	0.6940 ± 0.1061	0.5160 ± 0.0363
PTR	Right	1.3212 ± 0.1056**	1.1318 ± 0.0503	0.6849 ± 0.0982**	0.6065 ± 0.1065
SCC	–	1.1090 ± 0.0726	1.0465 ± 0.0528	0.8805 ± 0.1494	0.7519 ± 0.0905
TAP	Left	1.7470 ± 0.3212*	2.1038 ± 0.3082	0.7721 ± 0.2768	0.73465 ± 0.3890

Significant differences of AD and RD between RRMS patients and healthy controls are shown by FDR adjusted *p < 0.01 and highly significant differences by **p < 0.001.

## Discussion

In this study, the microstructural integrity was investigated on 19 NAWM regions in RRMS patients by combing the probabilistic lesion map and WM atlas method. We found widespread WM microstructural abnormalities revealed by DTI metrics, which Wallerian degeneration processes may cause. What is more, lower FA and higher MD, AD, and RD were found in RRMS patients in our study.

Diffusion tensor imaging technique has been widely used in MS-related researches for a long time, and WM tracts of MS patients in lesion and normal-appearing regions were proved significantly different from healthy controls. Most previous studies have used tract-based spatial statistics (TBSS) method, which have fewer misalignments but may be affected by partial volume effects due to the projected center/major tract. Although many WM atlas–based studies have been applied in the previous studies, few MS-related studies use this hybrid method. Here, WM atlas–based ROI analysis combined with the probabilistic lesion map was used to explore the integrity of the NAWM of RRMS. Since significant metabolic differences were found in frontal–parietal–occipital NAWM between RRMS patients and controls in our previous study in the same cohort, related 19 WM tracts were extracted from standard WM atlases for ROI analysis. The combination of the two methods may offer more information that may help to understand the pathophysiological mechanisms underlying MS of brain impairment and may improve the prediction of disease progression. Consistent with previous studies, there is a high probability that the lesions appear in the periventricular WM, and the generated probabilistic lesion map can reduce the side effects by using predefined ROIs or WM tracts. Nevertheless, due to applying the threshold, not all the lesion regions were excluded for the final binary mask, and they may make independent contributions to the DTI metrics.

In the current work, the NAWM in RRSM patients were found a widespread low FA trend with different degrees for all predefined NAWM tracts, and six regions achieved a statistically significant reduction, including the bilateral anterior corona radiata, entire CC, and right PTR. Significantly decreased FA values, which may be caused by demyelination, accumulation of inflammatory cells, and the axonal injury (Gass et al., 2001), have been detected in the majority of NAWM tracts, including the bilateral anterior corona radiata, body of CC, genu of the CC, and superior corona radiata. Axonal damage and demyelination in the CC are the key features in MS patients, which were directly proved by post-mortem tissue (Evangelou et al., 2000), DTI (Hasan et al., 2005) and MTR (Cercignani et al., 2001). In a recent study, Kim et al. (2017) found that the left tapetum of the CC showed lower FA and higher MD and RD values in MS patients using the TBSS method, but the results did not reach significance level. Our findings showed significantly decreased FA in the CC in RRMS patients, which was in line with previous findings (Hasan et al., 2005), who also found significant reductions in FA of the CC. Another study also found that aerobic fitness elevated the FA values of MS patients in left PTR, right anterior corona radiation, and the genu of the CC (Prakash et al., 2010). Different from a previous study, decreased FA in superior longitudinal fasciculus was not observed while decreased FA in posterior corona radiata and cingulum was reported (Preziosa et al., 2016). One possible reason is that the mean age of the subjects in previous research was much younger than that in our study, and another study found that the genu of the CC altered with age (Fan et al., 2019). However, Roosendaal et al. did not find decreased FA in the body of the CC (Roosendaal et al., 2009). The inconsistency of previous findings may be attributed to the different demographic characteristics and will motivate the MS community to explore the underlying mechanisms involved in DTI metrics.

Our findings that the MD values were elevated for all NAWM regions in RRMS patients have been widely confirmed (Senda et al., 2012; Kim et al., 2017) as well. The pathological reason for increased MD may be axonal loss, demyelination, edema, and remyelination. What is more, the MD values have been proved to correlate with the disease duration and expanded disability status scale scores (EDSS). A recent study reported a significant MD increase in many WM tracts in the RRMS patients compared with healthy controls, including the bilateral anterior corona radiata, genu of the CC, and superior corona radiata (Novo et al., 2018),which is in agreement with our findings. It should be noted that different from the high-significant decrease of the FA values in the CC, only the genu of the CC shows statistically decreasing MD indicating microscopic damages in RRMS patients in the current work. Also, the changes of the MD values in sub-region of the CC show inconsistent results in previous studies. For example, significantly increased MD in genu of the CC in MS patients has been found in Ge’s study (Ge et al., 2004) but not in other studies (Novo et al., 2018; Martínez-Heras et al., 2020) or our current study. Anderson’s group found that only the body of the CC showed significantly elevated MD values in MS patients; the genu and splenium of the CC were not (Andersen et al., 2018). As mentioned, it is still inconclusive depending on the existing studies.

We found that several ROIs showed significant differences between RRMS and controls in AD and RD. According to a previous study (Alexander et al., 2011), demyelination would lead to increased RD but unchanged AD. This study found decreased AD and increased RD in some NAWM tracts, including superior corona radiata, anterior corona radiata, external capsule, and PTR, which were consistent with previous studies (Preziosa et al., 2011; Liu et al., 2012). In theory, reduced AD, increased RD, or a combination of both will lead to reduced FA (Pierpaoli and Basser, 1996). Our study found FA decreased in anterior corona radiata, left posterior corona radiata, right PTR, and left superior corona radiata, which may be caused by increased RD. Meanwhile, the decreased FA in the other regions may be due to both metrics but not reduced AD.

The correlation between DTI metrics and MRS results may provide complementary clues to understanding the relationship between microstructure and neuronal metabolites (Goebell et al., 2006). Our previous study has found that the NAA/Cr ratio significantly decreases in the frontal and parietal–occipital NAWM (Sun et al., 2017) in the same cohort. However, only the CC shows significant difference changes with side-by-side visual comparison. Even so, Reid and Cheuk’s group have found a significant negative correlation between FA and glutamate+glutamine/creatine, and between N-acetylaspartate/creatine and RD in schizophrenia patients (Reid et al., 2016). Another group found that both N-acetylaspartate and apparent diffusion coefficient (ADC) significantly increased in WM lesion regions in RRMS patients after administration of the antidepressant fluoxetine (Sijens et al., 2008). Wijtenburg et al. (2013) found the NAA could explain the intersubjective FA variability in genu and splenium of the CC by comparing young and old healthy adults. The pathogenetic mechanisms of metabolites in NAWM of RRMS are still not clear, and their correlations with WM microstructure still need more studies in the future.

Many studies have investigated the correlations between DTI-derived metrics and MS symptoms. For example, using TBSS, cognitive impairment in RRMS was found to be strongly correlated with the WM alterations within the CC and PTR regions (Yu et al., 2012), which also show significantly lower FA and higher MD values in the current study. At the same time, the controversial results persisted, especially for relating with EDSS. Some studies reported a strong positive correlation between FA changes and EDSS (Liu et al., 2012; Shen et al., 2014), but others did not observe this correlation (Rashid et al., 2004a,b; Fink et al., 2010). Moreover, correlations between local DTI metrics and clinical symptoms, like visual acuity (Kolbe et al., 2012), motor disability (Prosperini et al., 2011), and memory (Audoin et al., 2007), were reported. Our findings of the abnormal NAWM tracts provided additional information and insights to understand the mechanisms underlying MS-associated symptoms. It should be kept in mind that the changes of the DTI metrics in most studies are not exactly the same as in WM tracts, thus the supportive explanations for each are much more complex. On the other hand, we must admit that the interpretation of DTI metrics changes is extremely challenging. Generally, the decreased FA and increased MD can be attributed to the demyelination and disruption integrity of WM. The AD changes are associated with axonal injury and losing fibers, whereas the RD changes are more related to the myelin content.

Although the relationship between the DTI metrics and the blood-oxygenation-level-dependent (BOLD) signal is complex, it is generally accepted that the microstructural integrity of WM is a prerequisite for normal BOLD activation and functional connectivity (Duerden et al., 2019; Huang et al., 2019; Jandric et al., 2021). Both increasing (Chiaravalloti et al., 2005) and decreasing (Sweet et al., 2006) BOLD activations were observed for MS patients in previous studies. For example, the significantly decreased FA and increased MD avulses found in ACR regions may correspond to the anterior functions of the default mode network (DMN). In addition, the NAWM changes in SCC and PTR regions may be related to the posterior part of DMN (Bonavita et al., 2011; Wu et al., 2020). Moreover, the significant MD and RD value changes in RRSM patients may be relevant to the DMN and dorsal attention networks (Huang et al., 2018). Our results may provide additional information to understand how the NAWM damages influence brain function.

### Limitations

There are several limitations in the present study. First, the correlation between the clinical symptoms, like EDSS, and the DTI metrics were not performed. Second, voxel’s locations of MRS in our previous study were not exactly the same as the NAWM tracts in the current study, thus the precise relationships between DTI metrics and MRS results were not explored in this pilot study. Third, although a probabilistic lesion map was generated to the greatest extent to compare the same tract, not exactly overlapped ROI voxels still existed and the results should be interpreted with caution.

## Conclusion

This study analyzed multiple diffusion indices in the NAWM tracts between RRMS patients and relatively healthy subjects, and extensive WM impairments were observed in RRMS patients using a hybrid method. To avoid the effects of obvious lesion regions, a probabilistic lesion map was generated using a semi-automatic method, which may be used in the future study toward getting rid of lesions. The RRMS patients showed statistically decreased FA and increased MD, AD, and RD in NAWM tracts compared with the control group. Our results using modified atlas-based method were partially in line with previous investigations of NAWM microstructural changes and may improve our understanding of the complex pathophysiology of RRMS.

## Data Availability Statement

The original contributions presented in the study are included in the article/supplementary material, further inquiries can be directed to the corresponding authors.

## Ethics Statement

This study was approved by the Local Institutional Ethics Committee on Human Research of the First Affiliated Hospital, and College of Clinical Medicine of Henan University of Science. The patients/participants provided their written informed consent to participate in this study.

## Author Contributions

JB and JL: conceptualization. HT and YL: design the study. HT: data acquisition and analysis. JS and FZ: project administration. ZH: methodology. All authors contributed to the article and approved the submitted version.

## Conflict of Interest

The authors declare that the research was conducted in the absence of any commercial or financial relationships that could be construed as a potential conflict of interest.

## Publisher’s Note

All claims expressed in this article are solely those of the authors and do not necessarily represent those of their affiliated organizations, or those of the publisher, the editors and the reviewers. Any product that may be evaluated in this article, or claim that may be made by its manufacturer, is not guaranteed or endorsed by the publisher.

## Footnotes

1
https://www.nitrc.org/projects/mricron

2
https://fsl.fmrib.ox.ac.uk/fsl/fslwiki/
